# Joint Contributions of Depression and Insufficient Sleep to Self-Harm Behaviors in Chinese College Students: A Population-Based Study in Jiangsu, China

**DOI:** 10.3390/brainsci13050769

**Published:** 2023-05-06

**Authors:** Yiwen Hua, Hui Xue, Xiyan Zhang, Lijun Fan, Yong Tian, Xin Wang, Xiaoyan Ni, Wei Du, Fengyun Zhang, Jie Yang

**Affiliations:** 1 School of Public Health, Southeast University, Nanjing 210096, China; 2 Department of Child and Adolescent Health Promotion, Jiangsu Provincial Center for Disease Control and Prevention, Nanjing 210009, China; 3 Institute of Child and Adolescent Health, Shanghai Municipal Center for Disease Control and Prevention, Shanghai 200336, China

**Keywords:** self-harm, sleep, depression, college students, China

## Abstract

Self-harm in young people is common, and previous studies have shown that insufficient sleep or depression was associated with self-harm. However, the joint association of insufficient sleep and depression with self-harm is unknown. We employed representative population-based data from the “Surveillance for Common Disease and Health Risk Factors Among Students in Jiangsu Province 2019” project. College students reported their self-harm behavior over the past year. Rate ratios (RRs) and corresponding 95% confidence intervals (CIs) for self-harm in relation to sleep and depression were modeled using negative binomial regression with a sample population as an offset, adjusting for age, gender, and region. The instrumental variable approach was used for the sensitivity analyses. Of the study population, approximately 3.8% reported self-harm behaviors. Students with sufficient sleep experienced a lower risk of self-harm than those with insufficient sleep. Compared with students with sufficient sleep and the absence of depression, the adjusted risk of self-harm was elevated 3-fold (1.46–4.51) in those reporting insufficient sleep in the absence of depression, 11-fold (6.26–17.77) in those with sufficient sleep and definite depression, and 15-fold (8.54–25.17) in those with both insufficient sleep and definite depression. The sensitivity analyses indicate that insufficient sleep remained a contributing risk factor for self-harm. Lack of sleep in young people is significantly associated with self-harm, particularly in the presence of depression. The provision of mental health care and attention to sleep deprivation are particularly important for college students.

## 1. Introduction

Youth self-harm is a growing problem in China and elsewhere in the world, possibly leading to suicide [1], with prevalence rates having increased over time [2,3]. Facing numerous challenges in fulfilling a variety of academic and social roles, college students frequently engage in various forms of self-injurious behaviors, experiencing an elevated risk of self-harm in comparison with the general population [4]. At least one in ten Chinese college students experience self-harm [5], similar to that in the United States (8%) [6], Belgium (10%) [7], and Japan (10%) [8] but lower than that in Canada (39%) [9], Indonesia (38%) [10], Iran (40%) [11], and Northern Ireland (19%) [12].

College life is a critical transition period for emerging adults between their late teens and early twenties, who are becoming more independent and face more profound academic and social challenges [5,6,7,8,9,10,11,12]. As the world’s largest developing country with the world’s largest higher education system, China had a total of 44.3 million college students in 2021 [13], ranking first in the world. While depressive symptoms and sleep problems are commonly observed among college students in China and elsewhere around the world [14], self-harm behaviors are also not rare phenomena [1,5,6,7,8,9,10,11,12]. It is widely accepted that youths who face depression or lack of sleep experience a higher risk of self-harm than their counterparts, for example, in the United State [6], Norway [15], United Kingdom [16], Australia [17], Indonesia [10], and Canada [18]. However, there is a lack of such reliable evidence in the Chinese setting.

Of identifiable risk correlates for self-harm, mood disorders, such as feeling depression or having a low mood or low self-esteem, were widely reported [1,8,12,19]. While mood disorders were prevalent in college students [8,12], multiple studies indicate that they had problems coping with these uncomfortable conditions [8,12,20]. The early onset of depression may correspond to an overall low psychosocial functioning in college students, leading to an increased risk for self-injurious behaviors to deal with such emotional turmoil [20].

Previous studies found that sleep deficiency was common in college students and was associated with self-injurious behaviors [21,22]. Poor sleep habits can possibly lead to serious mood disorders, as well as increase self-harm urges [21]. Lack of sleep was also related to impaired ability to perform academic and social tasks [14], as well as decreased mental flexibility [14]. Despite a bidirectional association between sleep and depression, research suggests psychosocial interventions to promote sleep for the health and well-being of youth, considering that insufficient sleep and depression are both highly prevalent at this critical life transition phase [14,22].

Self-harm behaviors are the interplay among many factors in addition to the aforementioned sleep and emotional factors. Several studies have reported that individuals with overweight/obesity experience a higher risk of self-harm, especially when they encounter social isolation and exclusion [23,24]. Excessive use of the Internet appeared to be an important risk factor for self-harm [25], and this is perhaps related to an intent to self-harm as well [19,26]. Smoking, alcohol consumption, and negative family environment (such as family discord, maladaptive parenting, and parental divorce) were also associated with self-harm [19]. In spite of the global consensus that the prevalence of self-harm decreases with age [19], the influence of sex on self-harm remains controversial. However, targeting the aforementioned risk factors to reduce self-harm in youths, multiple intervention programs have been developed and implemented but unfortunately with the results being inconclusive. The interplay among multiple factors perhaps explains why these factors are not amenable to interventions. While the existing literature reports different health outcomes in association with sleep and/or depression [1,2,19,21], few attempted to evaluate their independent and joint impact on self-harm in youths.

However, self-harm comprises heterogeneous behaviors, and recent reports indicate self-harm in association with depression or insufficient sleep in college students [10,15]. It is worth noting that depressive symptoms are closely related to sleep problems, although distinctions actually exist [14,21,22]. Given a dearth of evidence on such a complex bidirectional relationship, there is a need to quantify the joint contributions of depression and insufficient sleep to self-harm behaviors [21]. In this study, the research questions were as follows: (1) What is the prevalence of self-harm according to different sleep sufficiency classifications among college students in Jiangsu Province, China? (2) What demographic, lifestyle, and psychological factors are associated with self-harm? (3) Is self-harm associated with sleep insufficiency in the presence of depressive symptoms?

Considering that previous findings on self-harm in association with depression or sleep insufficiency can vary depending on where in the world subjects reside due, in part, to the differences in ethnicities and cultures, this study aimed to investigate the occurrence of self-harm among college students in Jiangsu Province, China, and to examine the relationship between sleep and self-injurious behaviors, especially in the presence of depression. These findings could increase awareness of ethic and cultural differences, which is vital to reduce the risk of self-harm in particular ethnic and cultural groups in similar settings.

## 2. Materials and Methods

### 2.1. Study Design

This was a cross-sectional study based on the “Surveillance for Common disease and Health Risk Factors Among Students in Jiangsu Province 2019” project.

### 2.2. Study Settings

This surveillance project was carried out in Jiangsu Province, located on the eastern coast of China. There are 167 universities or colleges in Jiangsu, providing tertiary education to approximately 1.7 million undergraduate students. Using a multistage, stratified, and randomized cluster sampling scheme, the project recruited a total of 13 universities and colleges, one from each prefecture-level administrative region in Jiangsu Province. Data were collected from September to November 2019, three months prior to the outbreak of the COVID-19 pandemic.

### 2.3. Participants

A total of 3209 students having spent 1 to 3 years in university or college participated in this study. All participants completed the self-reported questionnaires. All participants gave their written or verbal informed consent where appropriate. Ethics approval was obtained from the Institutional Review Board of Ethics committee of Jiangsu Provincial Center for Disease Prevention and Control.

### 2.4. Measurements

All variables in this study, except body mass index (BMI), were self-reported by the study participants. BMI was calculated by dividing the weight in kilograms by the square of height in meters, where the height (measured to the nearest 0.1 cm) and weight (measured to the nearest 0.1 kg) were measured by professionally trained research associates using the standardized equipment and procedures. The specific definitions of the variables are as follows.

#### 2.4.1. Exposures

The use of the Pittsburgh Sleep Inventory, Polysomnography, and/or other sleep trackers would perhaps deliver insight into the quality of sleep in the study population. However, these instruments were not available in the survey. Instead, sleep duration was adopted as a convenient proxy and assessed by asking the respondents: “How long did you sleep per night on average in the past week?”. Sleep insufficiency was routinely defined as fewer than 7 h of self-reported sleep per day [27]. The American Academy of Sleep Medicine and Sleep Research Society recommends a healthy adult have at least 7 h of sleep a day [28]. A meta-analysis showed that the average daily sleep duration of university students in China was 7.08 h (95% confidence interval: 6.84–7.32 h) [27]. Similarly, the Annual Sleep Report of China reported that the average daily sleep duration of university students was 7.04 h [29]. Accordingly, we categorized sleep duration as sufficient (≥7 h a day in the past week) or insufficient (<7 h).

To measure depression in the study population, we used the Center for Epidemiological Survey-Depression Scale (CES-D), which has been extensively employed in the literature. The CES-D scale is a 20-item self-reported instrument developed by Radloff [30], with 16 items assessing negative symptoms over the past week (e.g., “I felt lonely” and “I felt depressed”) and an additional 4 items measuring positive responses (e.g., “I enjoyed life” and “I was happy”). A 4-point response was applied to these 20 items, with 0 indicating “rarely or less than 1 day”; 1 indicating “some of the time or 1–2 days”; 2 indicating “a moderate amount of the time or 3–4 days”; and 3 indicating “most or all of the time or 5–7 days”. Scores for the four items pertinent to positive responses were reversely coded prior to score aggregation. The total CES-D score ranged from 0 to 60 points, the higher the score, the more probable depression. Using cut-off points appropriate for the Chinese setting [31], we further defined depression as none (0–15 points), probable depression (16–19 points), and definite depression (≥20 points), according to the Center for Epidemiological Survey-Depression Scale (CES-D Scale) [30]. This cut-off value of 16 points is widely accepted for classifying subjects with depression [30,31]. Allowing for “restless sleep” was included in the CES-D; however, we excluded it in the calculation of depressive scores.

In addition, we categorized gender as female or male; age group in years as <19 (*n* = 1014; proportion = 31.6%), 19–20 (1218; 38.0%), or >20 (977; 30.4%), corresponding to freshman, sophomore, and junior years at college; regions as Southern, Central, or Northern Jiangsu; boarding at school as yes or no; annual family income in the unit of 1000 Chinese Yuan as <50 (*n* = 888; proportion = 27.7%), 50–100 (1110; 34.6%), 101–200 (831; 25.9%), or >200 (380; 11.8%); paternal highest education attainment as primary school (*n* = 325; proportion = 10.1%), high school or equivalent (2362; 73.6%), or tertiary and above (522; 16.3%); maternal highest education attainment as primary school (*n* = 629; proportion = 19.6%), high school or equivalent (2221; 69.2%), or tertiary and above (359; 11.2%); having a core family background as yes or no; having siblings as yes or no; weight based on BMI as underweight (BMI < 18.5), normal (BMI: 18.5–23.9), or overweight/obesity (BMI > 24) [32]; and weekly physical exercises as sufficient (5+ times a week) or insufficient [33]. We categorized alcohol consumption, smoking, feeling hopeless, insomnia, or loneliness as binary, i.e., yes or no. We defined internet use as yes (greater than 4 h a day spent on the Internet for non-study-related learning purposes in the past week) or no according to Young’s internet addiction test [34] and the Core Information and Interpretation of Health Education for Chinese Adolescents (2018 Edition) [35].

#### 2.4.2. Main Outcomes

One question was asked in the survey with regards to self-harm, “Have you intentionally hurt yourself in the past 12 months, such as self-burning, self-cutting, head banging?” We have categorized the responses as binary, i.e., yes or no.

### 2.5. Data Collection

The “Surveillance for Common Disease and Health Risk Factors Among Students in Jiangsu Province 2019” project employed a multistage stratified, randomized cluster sampling scheme across all 13 prefecture-level administrative regions in Jiangsu province [36]. Additional information is available at the following website: http://www.moe.gov.cn/jyb_xxgk/gk_gbgg/moe_0/moe_8/moe_25/tnull_285.html (accessed on 4 April 2023). All eligible students selected from the 13 universities or colleges were invited to participated in this study. College coordinators identified eligible replacements for those who were unable to participate in the survey. Strict quality control programs were in place at every step during the field implementation to detect incorrect formats, invalid values, and missing data from the returned questionnaires. Additional call backs were carried out to investigate questionable data.

### 2.6. Data Analysis

We carried out all data analyses using SAS version 9.4 (SAS Institute, Cary, NC, USA) and STATA version 16.0 (StataCorp, College Station, TX, USA). A *p*-value less than 0.05 was set as statistically significant.

#### 2.6.1. Main Analysis

Numbers and proportions are presented for categorical variables. We modeled prevalence rates, rate ratios (RRs), and corresponding 95% confidence intervals (CIs) using negative binomial regression with a sample population as an offset, adjusting for age, gender, and region. We used the same adjustments to examine the relationship between each variable of interest (including gender, age group, regions, boarding at school, annual family income, paternal highest education attainment, maternal highest education attainment, core family background, having siblings, BMI, weekly physical exercises, alcohol consumption, smoking, internet use, insomnia, feeling hopeless, or loneliness) and self-harm. We further examined the relationship between sleep and self-harm in the presence of different levels of depression (i.e., without depression, probable depression, and definite depression) by introducing a sleep–depression interaction term in the modeling process.

#### 2.6.2. Sensitivity Analysis

Considering that cut-off values for sleep sufficiency vary across studies, we also carried out a sensitivity analysis using the threshold of sufficient sleep as at least 6 h a day to repeat the modeling process. We also assumed that the CES-D’s “restless sleep” measured a mental state of mind rather than sleep insufficiency and, therefore, we carried out additional analyses based on the depressive scores with the item “restless sleep” in the calculation.

#### 2.6.3. Instrumental Variable Analysis

Based on existing evidence in relation to sleep, depression, and self-harm behaviors, we hypothesized that the risk of self-harm in college students would be elevated by the joint contribution of insufficient sleep and depression. In order to make a causal inference, we employed the instrumental variable approach with demonstrated advantages for controlling unobserved sources of variability and potential measurement error in observational studies. Allowing for the control of an unmeasured confounding and potential reverse causal relationship between self-harm and insufficient sleep with depression, we further used daily dessert consumption as an instrumental variable for the sleep and depression interaction term, which was categorized as binary (i.e., yes or no) in the current study. Since dietary habits may affect sleep [37,38] and do not directly influence self-harm [19], the correlation and exogeneity of the instrumental variables were satisfied. We used an extended probit model considering that the instrumental variables were categorical.

## 3. Results

A total of 3209 college students with an age range of 15–26 years (representing 1.7 million of their peers in Jiangsu) provided complete information, where 51.1% (*n* = 1639) were females. Approximate 31.6% of the study population was aged under 19 years (Table 1). Students with insufficient sleep accounted for 26.7% of the study population. The average daily sleep duration was 7.37 (standard deviation: 1.29) hours. The prevalence rate of self-injurious behaviors was 3.79% (95%CI: 3.03–4.74%) in the study population. The prevalence rate of self-harm in college students with insufficient sleep (7.00%, 95%CI: 5.44–9.02%) was significantly higher than in those with sufficient sleep (2.69%, 95%CI: 2.01–3.59%).

Students with insufficient sleep were significantly more likely to report self-harm than those with sufficient sleep (RR = 2.59, 95%CI: 1.79–3.77). Self-harm seemed to be associated with a younger age group, non-core family background, maternal highest education attainment as tertiary and above, smoking, internet use, insomnia, depression, and feelings of hopelessness or loneliness, whereas an association with gender, regional residence, boarding at school, having siblings, obesity, physical exercises, and alcohol consumption showed little statistical significance in this study (Table 2).

Table 3 indicates that the risk of self-injurious behaviors increased with the severity of depression in college students, irrespective of insufficient or sufficient sleep. In the presence of definite depression, the rate ratio of self-harm was the greatest in students with insufficient sleep (RR = 14.66, 95%CI: 8.54–25.17). A sensitivity analysis indicates a similar pattern, where the risk of self-harm in students was related more strongly to sleep sufficiency in the presence of depression (Table 3 and Table 4).

In the instrumental variable analysis, the correlation of residual errors between self-harm and the focal exposure variables (i.e., sleep and depression) was significant (*p* < 0.01, irrespective of the different classifications of sleep sufficiency), which showed the existence of endogenous problems. The association between the instrument variable (i.e., dessert consumption) and the focal exposure variables (i.e., sleep and depression) was significant, indicating that there was no weak instrumental variable problem regardless of how sleep sufficiency was classified (*p* < 0.01). Table 5 shows the similar pattern of self-harm in association with sleep sufficiency in the presence of depression to the primary analysis. Although not statistically significant, the absolute risk of self-harm was the greatest in those who had insufficient sleep and definite depression (Table 5).

## 4. Discussion

### 4.1. Main Findings

We found that the independent and joint associations of insufficient sleep and depression with self-harm were significant. The coexistence of sleep insufficiency and depression was more strongly associated with the occurrence of self-harm than the presence of only sleep insufficiency or depression. College students with insufficient sleep experienced an elevated risk of self-harm, which is consistent with previous studies [21,22]. The strength of this sleep–self-harm association varied in the presence of depression. Self-injurious behaviors were reported 15 times as often in those with insufficient sleep and presence of definite depression than in those with sufficient sleep and absence of depression. While depression possibly mediated the relationship between sleep and self-harm [22], there is a dearth of studies on whether the risk of self-harm in youth would relate to the elevated levels of depression or to the aspects of their sleep, particularly at a population level. Depression and sleep problems are often concomitant [34], which explains the overlapping confidence intervals (Table 3 and Table 4). Researchers suggest that sleep problems are associated with an increased risk of self-harm and should be considered along with other variables, particularly psychological factors [21]. Specifically, this study demonstrates that when youths experience insufficient sleep, they are more likely to engage in self-injurious behaviors. This study also highlights the joint contribution of sleep and depression to self-harm in youths. Young people who do not get enough sleep may contend with depression and difficulties in emotional regulation, increasing the likelihood of self-harm [22]. These findings build on previous studies showing that a lack of sleep may be an important marker for self-injurious behaviors in youths. Current psychological interventions for poor sleep are effective, and it often seems less humiliating to seek help regarding sleep [21]. Mental health practitioners, educators, and policymakers should acknowledge that sleep/mood disturbances are a significant health concern in students and consider the role of university context factors in minimizing and preventing self-harm [14,21,22]. However, whether interventions to promote sleep health are effective in youth self-harm risk mitigation needs further study [14,21,22].

The relationship between depression and self-harm has been reported in previous studies [19,22]. In this study, depression seemed to have a greater impact on the risk of youth self-harm in comparison with insufficient sleep (Table 3). This may be related to the fact that negative emotions are proximal factors in self-harm. Individuals are prone to self-injurious behaviors when adverse psychological experiences occur but fail to deal with them [19]. An existing review hypothesizes that there might be a bidirectional association between sleep problems and depression [39]; for example, some mental disorders can lead to sleep problems, and the latter could exacerbate the psychiatric symptoms, which are more obvious in young people [22]. It also emphasizes that attention should be paid to interventions in sleep disorders whether before, during, or after the onset of depression [34]. Although lack of sleep was not collinear with depression in the current study, approximately 24% of college students with insufficient sleep reported probable or definite depression. This clearly reinforced the importance of providing mental health care in youths [14,20]; in doing so, physicians, teachers, and parents are expected to screen for mental disorders in youths and stay vigilant by keeping a watchful eye on those with borderline to serious sleep deprivation.

The risk of self-harm seemed to decline with age in this study, perhaps reflecting a psychosocial shock by various life-changing factors during the freshman stage. With the increase in age and experience, social adaptability and stress resistance gradually improves [2]. In addition to age, factors such as living in non-core families, maternal highest education attainment as tertiary and above, smoking, internet use, and insomnia, as well as feelings of hopelessness or loneliness, were associated with youth self-harm, which is consistent with previous findings [1,19]. Of these identified risk factors, living in a non-core family often indicates the absence of a parent in the family composition, which might result in maladaptive parenting and, therefore, affect youth mental health and even trigger self-injurious behaviors. The Internet can not only facilitate access to online emotional support but also expose vulnerable users to distressing or harmful content that might risk triggering or exacerbating their self-harm behaviors. Given that the majority of university or college students are exposed to extensive use of the Internet, how to regulate the appropriateness of internet use to take advantage of its merits and avoid its negative impacts has become an urgent issue. Insomnia, one of the manifestations of insufficient sleep, has been reported in association with self-harm, and this association may be mediated by psychological factors. Feeling hopeless or feeling lonely appeared to be a plausible factor for self-harm in youth. However, the current study did not have any causal information, for example, whether there were acute life or school events occurring beforehand and, therefore, we are unable to elucidate processes of such mental disturbance contributing to self-harm in youth. Nonetheless, informed countermeasures to reduce youth self-injurious behaviors are warranted, including the provision of social support networks and/or capacity-building mechanisms for vulnerable students to cope with acute life or school events.

### 4.2. Implications for Practice and Policy

Recently, the Ministry of Education of the People’s Republic of China issued the notice on Further Strengthening the Sleep Management in Primary and Secondary School Students, prescribing school start and finish times and hours of sleep per day [40]. Considering that a lack of sleep is also common in college students and leads to unfavorable outcomes, including elevated risks of mood disorders [21], new sleep policies targeting residential students at colleges can be developed in addition to existing policies to improve mental health care [41]. Moreover, mental health services for college students are expected to be strengthened and encouraged to target freshmen students, such as providing available assistance or counseling services, cultivating right ways to process emotions [3], and enhancing abilities to solve social and personal problems [13,14,22,41]. Candidate strategies [19,20] to improve mental wellness in university or college students could consider (1) raising awareness of mental health support facilities and addressing social stigma to reduce barriers to service utilization; (2) creating and designing an on-campus culture for well-being and advocating for a more friendly family–college–community collaboration using mass media and other communications; (3) investing digital mental health ambulatory services and providing 24 h psychological counselling services online; and (4) establishing an on-campus responsive and prompt crisis reaction force, where university or college staff and faculty should be empowered with capacity-building skills and training to carry out timely interventions in response to crisis in students.

### 4.3. Strengths and Limitations

This study is one of its kind, specifically targeting the joint contributions of depression and insufficient sleep to self-harm behaviors in college students. This is of paramount importance, because with growing concerns of sleep deprivation linked to mental health in college students, they experience a lack of sleep and spend too long on mobile devices and desktop computers, as well as social activities. Increasing evidence also shows that depression in young adults might worsen their sleep disruption. This study estimated the independent roles of depressions and sleep insufficiency, as well as their joint role, in association with self-harm behaviors in Chinese college students, and it reveals that they have different detrimental impacts on the risk of self-harm. Although depression in comparison with insufficient sleep showed a marginally greater independent impact on the risk of self-harm, their joint contribution demonstrates the importance of sleep problems in the presence of depression and provides new avenues for effective sleep-related intervention strategies, such as maintaining a consistent sleep schedule and keeping relatively brief nap habits to reduce youth self-harm, especially among those with depression who could be successfully treated.

In the current study, we evaluated the joint role of depression and sleep insufficiency in association with self-harm behaviors in Chinese college students. A range of factors, such as parental education background, family income, and coexisting disease burden, might likely contribute to the risk of youth self-harm. Although we attempted to investigate these by controlling for age, sex, and region in the modeling process, the results should be interpreted with care, allowing for the cross-sectional nature of the study. Future prospective data collection may have the facility for multivariate investigation. With the development of precise risk prediction on the horizon, we could perhaps achieve more conclusive indications. Nevertheless, our findings provide informative recommendations for policymakers and youth health advocates to consider appropriate countermeasures to reduce the risk of self-harm in college students and their peers in similar settings without ignoring that these emerging adults with multiple risk factors would possibly experience an elevated risk of self-harm.

However, several limitations should be noted when interpreting the results. First, the cross-sectional nature of this investigation cannot confirm any causal relationship of sleep and depression to self-harm in youth. Due to the lack of genetic variables, we could not perform Mendelian randomization to explore the issue of endogeneity. However, to account for unmeasured confounding, we adopted the instrumental variable approach and found that the association between insufficient sleep and self-harm was somewhat robust. Nonetheless, a future cohort design is warranted to evaluate the longitudinal sleep–self-harm association. Second, the occurrence of self-harm is subject to social stigma, as some students might not be willing to provide information on their self-injurious behaviors and, therefore, its burden in college students could be underestimated. Third, for practical reasons, this study employed a self-report instrument for the confirmation of depression instead of clinical interviews, clearly representing another limitation. Depression and sleep duration were based on past-week conditions, while self-harm was reported for the past year (ideally self-harm should also be reported as having occurred in the past week). However, given that repetition of self-harm is common [19], the possibility that the behavior occurred in the past week cannot be ruled out. Our findings should be interpreted with caution. Fourth, sleep duration can change from person to person. We considered the American Academy of Sleep Medicine and Sleep Research Society criteria and applied different cut-off values for the sensitivity analyses. Although we observed few material changes, multidimensional data for sleep quality would perhaps present a more comprehensive measurement to inform potential intervention strategies to help students to achieve better sleep. Future studies can employ scales, for instance, the Pittsburgh Sleep Quality Inventory and Mini-Sleep Questionnaire, to assess sleep patterns, as well as psychometric properties in late adolescence. Fifth, while the study population was recruited from universities or colleges, one from each prefecture-level municipalities in Jiangsu Province, the representativeness would be impacted by variation in access to the sample. Given the project employed a multistage, randomized sampling scheme, the current findings indicate a somewhat robust demonstration of youth self-harm in Jiangsu Province. However, given that Jiangsu Province is an affluent and populous coastal area in China, the results should be interpreted with care under circumstances of different study settings. Sixth, the current study only modeled a handful of contextual factors, such as regions, whether boarding at school, and different types of family compositions, lacking consideration of other contextual factors, such as college-specific resources and cultures, in response to academic and emotional crisis in university and college students. Future studies can account for a variety of contextual factors with the possibility of providing additional insight into the design of programs for the prevention and control of self-harm behaviors.

## 5. Conclusions

During their emerging adulthood, college students go through an important transitional period, perhaps embedded with a variety of mental health challenges. College life is also a crucial stage for these emerging adults in their late adolescence to develop and improve their mental well-being; however, self-harm is unfortunately accentuated among them when independently dealing with different study and life struggles. To reduce youth self-harm, lack of sleep requires close attention in the mental health community, particularly in the presence of depression. The provision of timely mental health care and other forms of psychosocial support services on campus is also of paramount importance for college students facing a crisis in adapting to unexpected disruptions. Continuing investments are necessary to promote streamlined two-way communication with regards to both regular outreach and rapid response in crisis management systems. Future studies are encouraged to evaluate the comparative effectiveness of different risk management and mitigation strategies to avoid repeated self-harm and improve mental well-being in a rapidly changing academic environment.

## Figures and Tables

**Table 1:** Prevalence rate of self-harm among 3209 college students in relation to demographic, lifestyle, and psychological factors by sleep status.

Variables	Insufficient Sleep			Sufficient Sleep		
	N	n	Rate (95%CI)	N	n	Rate (95%CI)
**Age**						
<19	344	27	7.85 (5.38–11.45)	670	22	3.31 (1.99–5.49)
19–20	329	25	7.60 (5.13–11.25)	889	26	2.89 (1.85–4.52)
>20	184	8	4.35 (2.17–8.69)	793	15	1.89 (1.14–3.14)
**Gender**						
Male	384	28	6.87 (4.11–11.50)	1186	35	2.95 (2.12–4.11)
Female	473	32	6.77 (4.78–9.57)	1166	28	2.40 (1.45–3.96)
**Region**						
Southern Jiangsu	295	23	7.80 (5.18–11.73)	943	28	2.97 (2.05–4.30)
Central Jiangsu	189	10	4.99 (2.23–11.13)	528	14	2.67 (1.53–4.64)
Northern Jiangsu	373	27	7.24 (4.96–10.56)	881	21	2.48 (1.42–4.34)
**Boarding**						
Yes	815	-	6.87 (5.29–8.93)	2272	-	2.55 (1.96–3.34)
No	42	-	13.56 (3.21–57.37)	80	-	6.25 (2.60–15.02)
**Annual family income in the unit of 1000 Chinese Yuan**						
<50	239	21	8.79 (5.73–13.48)	649	22	3.39 (2.23–5.15)
50–100	305	20	6.96 (4.16–11.66)	805	15	1.86 (1.12–3.09)
101–200	219	10	4.57 (2.46–8.49)	612	15	2.85 (1.51–5.40)
>200	94	9	9.57 (4.98–18.40)	286	11	3.85 (2.13–6.95)
**Paternal highest education attainment**						
Primary school	95	8	8.42 (4.21–16.84)	230	-	2.17 (0.90–5.22)
High school or equivalent	642	40	6.23 (4.57–8.49)	1720	-	2.09 (1.51–2.90)
Tertiary and above	120	12	10.00 (4.63–15.37)	402	-	5.47 (3.60–8.31)
**Maternal highest education attainment**						
Primary school	157	11	7.01 (3.88–12.65)	472	12	2.54 (1.44–4.48)
High school or equivalent	606	38	6.27 (4.56–8.62)	1615	37	2.29 (1.66–3.16)
Tertiary and above	94	11	11.70 (6.48–21.13)	265	14	5.27 (3.10–8.96)
**Core family**						
Yes	406	19	4.40 (2.51–7.72)	1094	23	2.10 (1.40–3.16)
No	451	41	9.09 (6.69–12.35)	1258	40	3.17 (2.31–4.36)
**Having siblings**						
No	481	32	6.38 (4.20–9.71)	1320	32	2.43 (1.67–3.53)
Yes	376	28	7.45 (5.14–10.79)	1032	31	3.00 (2.10–4.31)
**Physical exercise, weekly**						
Insufficient	692	47	6.79 (5.10–9.04)	1840	48	2.63 (1.90–3.64)
Sufficient	165	13	7.88 (4.57–13.57)	512	15	2.93 (1.77–4.86)
**BMI**						
Underweight	102	8	7.84 (3.92–15.68)	327	-	1.54 (0.60–3.93)
Normal	512	37	7.23 (5.24–9.97)	1443	-	2.84 (2.09–3.87)
Overweight/obesity	243	15	6.04 (3.42–10.67)	582	-	2.92 (1.82–4.70)
**Alcohol consumption**						
No	459	24	5.23 (3.50–7.80)	1277	32	2.60 (1.73–3.89)
Yes	398	36	9.05 (6.52–12.54)	1075	31	2.88 (1.97–4.21)
**Smoking**						
No	735	45	6.12 (4.57–8.20)	2020	51	2.53 (1.90–3.35)
Yes	122	15	12.30 (7.41–20.39)	332	12	3.68 (1.97–6.86)
**Internet use**						
No	782	53	6.78 (5.18–8.87)	2199	49	2.23 (1.65–3.01)
Yes	75	7	9.33 (4.45–19.58)	153	14	9.15 (5.42–15.45)
**Depression**						
None	653	23	3.52 (2.34–5.30)	1980	26	1.31 (0.86–1.98)
Probable	73	10	13.70 (7.37–25.46)	145	6	4.14 (1.86–9.21)
Definite	131	27	20.37 (13.56–30.61)	227	31	14.02 (8.91–22.06)
**Insomnia**						
No	397	12	3.00 (1.64–5.50)	1078	12	0.94 (0.53–1.67)
Yes	460	48	10.34 (7.59–14.07)	1274	51	4.91 (3.39–7.10)
**Feeling hopeless**						
No	683	21	3.07 (2.00–4.72)	2078	28	1.35 (0.93–1.95)
Yes	174	39	22.42 (16.38–30.68)	274	35	12.77 (9.17–17.79)
**Loneliness**						
No	295	-	1.69 (0.70–4.08)	1067	8	0.75 (0.37–1.50)
Yes	562	-	9.79 (7.51–12.75)	1285	55	4.29 (3.05–6.04)
**Total**	857	60	7.00 (5.44–9.02)	2352	63	2.69 (2.01–3.59)

Cells with a number ≤ 5 and its relational cell are represented with a “-” sign.

**Table 2:** Adjusted rate ratios (RRs) and 95% confidence intervals (CIs) for self-harm according to demographic, lifestyle, and psychological factors.

Variable	Adjusted RR (95%CI)
**Age, years**	
>20	Reference
19–20	1.78 (1.08–2.93)
<19	2.05 (1.25–3.38)
**Gender**	
Male	Reference
Female	0.90 (0.63–1.29)
**Region**	
Southern Jiangsu	Reference
Central Jiangsu	0.87 (0.53–1.41)
Northern Jiangsu	0.99 (0.66–1.47)
**Boarding**	
Yes	Reference
No	1.97 (0.97–4.01)
**Annual family income in CNY 1000**	
<50	Reference
50–100	0.63 (0.40–0.99)
101–200	0.60 (0.36–0.98)
>200	1.05 (0.62–1.79)
**Paternal highest education attainment**	
Primary school	Reference
High school or equivalent	0.77 (0.43–1.39)
Tertiary and above	1.56 (0.82–2.97)
**Maternal highest education attainment**	
Primary school	Reference
High school or equivalent	0.89 (0.56–1.43)
Tertiary and above	1.84 (1.04–3.25)
**Core family**	
Yes	Reference
No	1.69 (1.16–2.45)
**Having siblings**	
No	Reference
Yes	1.24 (0.87–1.79)
**BMI**	
Underweight	Reference
Normal	1.31 (0.73–2.35)
Overweight/obesity	1.24 (0.65–2.38)
**Physical exercise, weekly**	
Insufficient	Reference
Sufficient	1.04 (0.68–1.61)
**Alcohol consumption**	
No	Reference
Yes	1.44 (0.99–2.09)
**Smoking**	
No	Reference
Yes	1.78 (1.12–2.80)
**Sleep**	
Sufficient	Reference
Insufficient	2.59 (1.79–3.77)
**Internet use**	
No	Reference
Yes	2.80 (1.75–4.48)
**Depression**	
None	Reference
Probable	4.04 (2.26–7.21)
Definite	8.69 (5.81–12.99)
**Insomnia**	
No	Reference
Yes	4.64 (2.97–7.26)
**Feeling hopeless**	
No	Reference
Yes	9.39 (6.54–13.48)
**Loneliness**	
No	Reference
Yes	6.24 (3.51–11.08)

RRs were adjusted for age, gender, and region.

**Table 3:** Adjusted rate ratios (RRs) and 95% confidence intervals (CIs) for self-harm in relation to sleep insufficiency in the presence of depression (Total CES-D score = 57) ^c^.

Variable	Adjusted RR ^a^ (95%CI)	Adjusted RR ^b^ (95%CI)
**Sufficient sleep**		
No depression	Reference	Reference
Probable depression	3.30 (1.35–8.01)	3.49 (1.79–6.80)
Definite depression	10.55 (6.26–17.77)	9.00 (5.93–13.66)
**Insufficient sleep**		
No depression	2.56 (1.46–4.51)	5.40 (2.51–11.61)
Probable depression	10.01 (4.80–20.85)	18.20 (7.14–46.43)
Definite depression	14.66 (8.54–25.17)	21.10 (10.55–42.22)

RRs were adjusted for age, gender, and region. ^a^ Sufficient sleep was defined as at least 7 h a day. ^b^ Sufficient sleep was defined as at least 6 h a day. ^c^ The depression score was recalculated by excluding the item “restless sleep” in the original CES-D scale.

**Table 4:** Adjusted rate ratios (RRs) and 95% confidence intervals (CIs) for self-harm in relation to sleep insufficiency in the presence of depression (Total CES-D score = 60) ^c^.

Variable	Adjusted RR ^a^ (95%CI)	Adjusted RR ^b^ (95%CI)
**Sufficient sleep**		
No depression	Reference	Reference
Probable depression	4.79 (2.15–10.68)	4.16 (2.17–7.96)
Definite depression	10.23 (6.00–17.45)	9.03 (5.92–13.77)
**Insufficient sleep**		
No depression	2.55 (1.41–4.59)	5.10 (2.26–11.50)
Probable depression	9.14 (4.08–20.59)	18.01 (6.35–51.03)
Definite depression	16.16 (9.47–27.59)	24.02 (12.25–46.08)

RRs were adjusted for age, gender, and region. ^a^ Sufficient sleep was defined as at least 7 h a day. ^b^ Sufficient sleep was defined as at least 6 h a day. ^c^ The depression score was recalculated by including the item “restless sleep” in the original CES-D scale.

**Table 5:** Joint association of sleep and depression with self-harm under different sleep sufficiency classifications in the Instrumental Variable analysis.

Variable ^a^	Adjusted Risk of Self-Harm			
	≥7 h a Day as Sufficient Sleep		≥6 h a Day as Sufficient Sleep	
	Eprobit β (95%CI) ^b^	AME β (95%CI) ^c^	Eprobit β (95%CI) ^b^	AME β (95%CI) ^c^
**Sufficient sleep**				
No depression	Reference	Reference	Reference	Reference
Probable depression	2.13 (0.62, 3.64) **	0.03 (−0.16, 0.22)	2.55 (1.65, 3.44) ***	0.04 (−0.19, 0.26)
Definite depression	2.94 (1.55, 4.33) ***	0.12 (−0.28, 0.53)	3.23 (2.55, 3.90) ***	0.13 (−0.17, 0.43)
**Insufficient sleep**				
No depression	1.51 (0.35, 2.67) *	0.02 (−0.10, 0.14)	2.42 (1.64, 3.20) ***	0.07 (−0.20, 0.33)
Probable depression	2.29 (1.23, 3.35) ***	0.12 (−0.19, 0.43)	2.84 (2.33, 3.36) ***	0.28 (−0.12, 0.67)
Definite depression	2.58 (1.61, 3.56) ***	0.18 (−0.19, 0.56)	2.91 (2.48, 3.34) ***	0.31 (−0.04, 0.66)

* *p* < 0.05, ** *p* < 0.01, *** *p* < 0.001. ^a^ Depression categories were reclassified excluding the item “restless sleep” in the original CES-D scale. ^b^ The β coefficients from all extended probit regression (Eprobit) models were further adjusted for age, gender, and region. ^c^ The estimated average marginal effects (AMEs) and associated 95% confidence interval (CI) of sleep in the presence of depression on the probability of self-harm were also reported.

## Data Availability

Due to the nature of this research, participants in this study did not agree for their data to be shared publicly, so data are not available.
